# Predictors of Postpartum Glucose Tolerance Testing in Italian Women with Gestational Diabetes Mellitus

**DOI:** 10.1155/2013/182505

**Published:** 2013-07-17

**Authors:** Carmelo Capula, Eusebio Chiefari, Anna Vero, Stefania Iiritano, Biagio Arcidiacono, Luigi Puccio, Vittorio Pullano, Daniela Foti, Antonio Brunetti, Raffaella Vero

**Affiliations:** ^1^Complex Operative Structure Endocrinology-Diabetology, Hospital Pugliese-Ciaccio, 88100 Catanzaro, Italy; ^2^Department of Health Sciences, University “Magna Græcia” of Catanzaro, 88100 Catanzaro, Italy

## Abstract

Postpartum screening is critical for early identification of type 2 diabetes in women previously diagnosed with gestational diabetes mellitus (GDM). Nevertheless, its rate remains disappointingly low. Thus, we plan to examine the rate of postpartum glucose tolerance test (ppOGTT) for Italian women with GDM, before and after counseling, and identify demographic, clinical, and/or biochemical predictors of adherence. With these aims, we retrospectively enrolled 1159 women with GDM, in Calabria, Southern Italy, between 2004 and 2011. During the last year, verbal and written counseling on the importance of followup was introduced. Data were analyzed by multiple regression analysis. A significant increase of the return rate was observed following introduction of the counseling [adjusted odds ratio (AOR) 5.17 (95% CI, 3.83–6.97), *P* < 0.001]. Interestingly, previous diagnosis of polycystic ovary syndrome (PCOS) emerged as the major predictor of postpartum followup [AOR 5.27 (95% CI, 3.51–8.70), *P* < 0.001], even after stratification for the absence of counseling. Previous diagnosis of GDM, higher educational status, and insulin treatment were also relevant predictors. Overall, our data indicate that counseling intervention is effective, even if many women fail to return, whereas PCOS represents a new strong predictor of adherence to postpartum testing.

## 1. Introduction

Gestational diabetes mellitus (GDM) is historically defined as “any degree of glucose intolerance with onset or first recognition during pregnancy” [1]. Incidence of GDM is increasing worldwide for recent trends in obesity and advancing maternal age, significantly contributing to increased overall health-care and economic costs [2, 3]. Approximately 7% of all pregnancies are complicated by GDM, resulting in more than 200,000 cases annually [4–7]. Women with GDM are at high risk for short pregnancy complications, such as gestational hypertensive disorders, fetal macrosomia, shoulder dystocia, and cesarean delivery [8–10]. In addition, GDM constitutes a high risk for future type 2 diabetes mellitus (DM) and cardiovascular disease [11–13]. In particular, women with GDM, even with mild glucose intolerance, have up to seven times more risk of developing type 2 DM compared to women with normoglycemic pregnancies [13–15], thus justifying recently recommended tighter diagnostic criteria for GDM [16]. 

Based on the compelling evidence that lifestyle intervention can effectively prevent or delay the development of type 2 DM [17–19], early identification of women at high risk of diabetes is very important. In this regard, postpartum is a critical period for early diagnosis and for planning prevention and intervention strategies [20–24]. Consistently, the Fifth International Workshop-Conference on Gestational Diabetes Mellitus Panel recommends that women with GDM have a 2-hour 75 gr oral glucose tolerance test (OGTT) at 6 weeks to 12 weeks postpartum [25]. Nevertheless, the majority of women fail to return for postpartum oral glucose testing (ppOGTT) [26–30]. Many reasons have been proposed for such lack of compliance, including poor communication between obstetrician and primary care provider, some confusion over the current guidelines, poor bridging from antepartum to postpartum care, lack of patient awareness, and a certain lack of interest in patient's personal health [30–32]. Therefore, in the last years, many tools have been tested to improve the coverage of screened women, including education intervention among women diagnosed with GDM, automated orders to primary care providers, and telephone and e-mail reminder messages to patients [33–37].

Based on this background, we designed a retrospective study in an Italian population in order to examine adherence rate to ppOGTT and evaluate the efficacy of educational intervention, identifying what are the predictor factors of compliance.

## 2. Methods 

### 2.1. Study Population

6770 Caucasian pregnant women attended the Hospital “Pugliese-Ciaccio” in Catanzaro (Calabria, Southern Italy), from January 2004 to December 2011, for GDM screening. Out of these, 1159 (17.1%) were diagnosed with GDM, following the current criteria [16, 38]. All consecutive pregnant women with GDM were included, except those with preexisting diabetes, as defined by ADA criteria [1]. Anamnestic information was obtained regarding age, instruction level, parity, previous GDM, family history of diabetes, self-reported prepregnancy weight, and previous polycystic ovary syndrome (PCOS), as diagnosed according to the Rotterdam criteria [39].

To promote adherence to ppOGTT, after January 2011, verbal and written counseling was given to 247 women at 35–40 weeks of pregnancy: they were informed about the increased risk for type 2 DM and about the risks to start a new pregnancy in the presence of glucose intolerance. Then, pregnant ones were provided with a simple handout illustrating the risk of GDM, follow-up recommendations, and suggestions for a correct lifestyle, as type 2 DM preventive strategy. The study was approved by the local ethics committee.

### 2.2. Statistical Analysis

The nonparametric Mann-Whitney *U* test was used for comparisons of continuous variables and the 2-tailed Fisher exact test was used for comparisons of proportions. Generally, a significance level of 0.05 was set for a type I error in all analyses. Logistic regression analysis was used to evaluate individual effects of each patient's categorical characteristic, including counseling as possible predictor of ppOGTT postpartum testing. Odds Ratios (ORs) with 95% confidence bounds were calculated. Linear regression analysis was employed to test the association of continue variables with adherence to followup. Each quantitative trait was tested for normality using the Shapiro-Wilk normality test and, when required, it was log-transformed. Data were analyzed with the SPSS 20.0 software. Post hoc statistical power calculations were performed using G∗Power software 3.1.3 (Franz Faul, Kiel University, Kiel, Germany).

## 3. Results

1159 women were diagnosed as affected by GDM. Out of these, 374 (32.3%) underwent postpartum diabetes screening whereas the remaining 785 (67.7%) did not.  Table 1 summarizes demographic, anthropometric, clinical, and biochemical characteristics of both cohorts. Age at diagnosis, prevalence of previous GDM, instruction level, PCOS, fasting plasma glucose, and prevalence of insulin treatment were higher in the group adherent to followup compared with the group not adherent to followup. No difference was observed for prepregnancy BMI, familiarity for type 2 DM, and parity (Table 1).

### 3.1. Counseling Effectiveness

**Figure 1:**
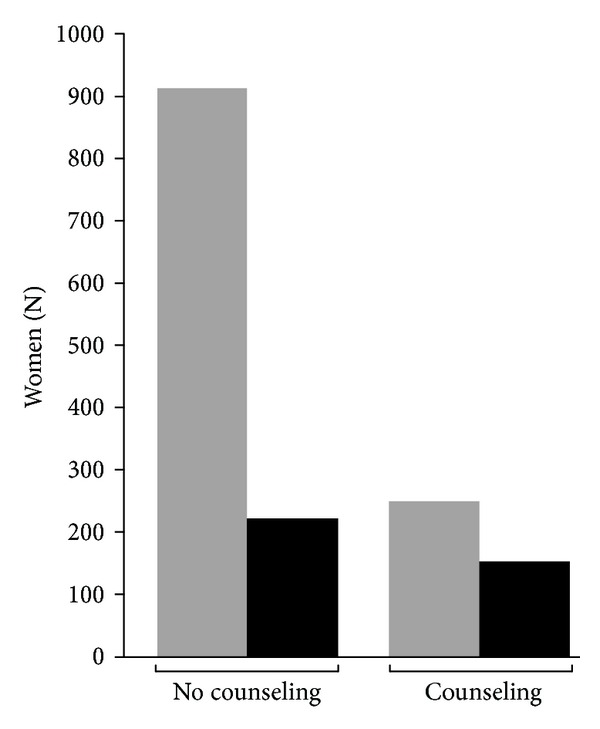
Adherence to ppOGTT in women with no counseling or after counseling. Gray bars, total women. Black bars, compliant women.

To test the effectiveness of verbal and written counseling, we compared the return rate of women receiving this intervention (62.3%, 154/247) with that of women who did not receive this intervention (24.1%, 220/912) (Figure 1). After adjustment for possible confounders, logistic regression analysis showed a strong association between counseling intervention and adherence to ppOGTT [adjusted OR (AOR) 5.17 (95% CI, 3.83–6.97), *P* < 0.001] (Table 2). After suitable stratifications, adherence to followup appeared higher among women with middle/high educational status [AOR 5.25 (95% CI, 3.60–7.65), *P* < 0.001] with respect to women with lower educational status [AOR 4.08 (95% CI, 2.26–7.38), *P* < 0.001]; higher among women ≥30 years old [AOR 5.64 (95% CI, 3.90–8.14), *P* < 0.001], compared with younger women [AOR 4.66 (95% CI, 2.71–8.01), *P* < 0.001]; higher among multigravid women [AOR 5.91 (95% CI, 3.93–8.89), *P* < 0.001] with respect to primigravidas [AOR 4.46 (95% CI, 2.86–6.94), *P* < 0.001] (Table 2). In all cases statistical power exceeded 95%.

### 3.2. Other Predictors of ppOGTT

To identify other predictors of ppOGTT, clinical and biochemical parameters that may influence screening rates were employed into a logistic regression analysis (Table 3(a)). As expected, a previous diagnosis of GDM, as well as a higher educational status, strongly correlated with a better adherence to ppOGTT [AOR 4.82 (95% CI, 3.17–7.33), *P* < 0.001, and AOR 4.06 (95% CI, 3.02–5.45) *P* < 0.001, resp.] (Table 3(a)). Also, insulin treatment during pregnancy was a predictor of follow-up adherence [AOR 2.32 (95% CI, 1.76–3.05), *P* < 0.001]. A mild association between prepregnancy BMI and ppOGTT was observed among overweight/obese women when compared with lean women [AOR 1.41 (95% CI, 1.09–1.81), *P* = 0.008] (Table 3(a)). Interestingly, a previous diagnosis of PCOS emerged as the major predictor of postpartum evaluation of glucose tolerance in women with GDM [AOR 5.27 (95% CI, 3.36–8.27), *P* < 0.001] (Table 3(a)). No association was observed with other factors, such as family history of type 2 DM and parity (Table 3(a)). To exclude influence of counseling intervention on detected predictors, a multiple regression analysis, after stratification for the absence of counseling, has been performed. As shown in Table 3(a), no substantial change emerged for any factor, indicating that their predicting effect was independent of counseling.

When continuous variables were tested, significant association of ppOGTT was observed with earlier diagnosis of GDM (*P* < 0.001) and age (*P* < 0.001) and fasting plasma glucose (*P* = 0.027) at diagnosis of GDM (Table 3(b)). For all these traits statistical power exceeded 95%.

## 4. Discussion

Herein, we investigated the rate of ppOGTT in Calabrian women, a Southern Italian population characterized by higher prevalence of GDM, type 2 DM, and obesity, as compared to the entire Italian population (http://www.istat.it). As reported in this study, pregnant women who underwent prepartum counseling had a significantly better follow-up adherence rate than those who had no counseling. Counseling was more effectiveness in older women and in women with higher educational level and with previous pregnancies. This is consistent with a major degree of awareness of health risks in these women with respect to younger women, women with low educational status, or primigravidas. For the first time, previous diagnosis of PCOS emerged as the stronger predictor of ppOGTT, with a similar and independent strength with respect to counseling. As an explanation for this, women with PCOS might be more willing to accept medical recommendations, since they often experience unpleasant clinical manifestations such as menstrual irregularity, infertility, and hirsutism. Moreover, because patients with PCOS are often treated with metformin, a widely used antidiabetic drug, these women might be more aware of the risk of type 2 DM and might have more contacts within the health-care system.

In a similar way to other reports [26, 27, 40], our results indicate that the most compliant ones were older women, with a previous diagnosis of GDM, higher educational levels, overweight, or obese and those with insulin treatment, higher FPG, and an early diagnosis of GDM. It is plausible that these women may have had greater awareness of their risk for becoming diabetics. 

## 5. Conclusions

In summary, we show that counseling is an effective, inexpensive, and simple tool in increasing ppOGTT rates for women with GDM. Moreover, patients with a previous diagnosis of PCOS were found to be significantly associated with a higher compliance rate for this test. Therefore, counseling can be more effectively targeted based on these observations. Further studies are needed to see whether our findings can be generalized to other populations.

## Figures and Tables

**Table 1:** General characteristics of the GDM population.

	Followup (*n* = 374)	No followup (*n* = 785)	*P* value
Race	Caucasian	Caucasian	—
Age (yr), median	36	33	<0.001
Range	18–48	18–46	—
BMI (kg/m^2^), median	25.3	24.5	0.175
Range	19–44.8	17.6–45	—
Familiarity for type 2 DM (*n*)	270	535	0.150
Previous GDM	72	45	<0.001
Parity (*n*), median	2	2	0.177
Range	1–5	1–6	—
>1	207	438	0.900
Educational status			
Low	71	384	<0.001
Middle	256	356	<0.001
High	47	45	<0.001
FPG (mg/dL), median	94	92	0.020
Range	70–121	72–126	—
Insulin treatment	147	253	0.021
PCOS	72	37	<0.001

Mann-Whitney U test was used for comparison of continuous traits. 2-tailed Fisher exact test was used for comparison of proportions. SD: standard deviation; BMI: body mass index; low educational status: primary school; middle educational status: secondary school; high educational status: university degree; FPG: fasting plasma glucose; PCOS: polycystic ovary syndrome.

**Table 2:** Effect of counseling on follow-up adherence.

	Stratification	Followup (*n* = 374)	No followup (*n* = 785)	OR (95% CI)	*P* value	Statistical power
Counseling (*n*)	None	154	93	5.17 (3.83–6.97)	<0.001	>95.0%
No counseling (*n*)		220	692			

Counseling (*n*)	Low educational status	24	47	4.08 (2.26–7.38)	<0.001	>95.0%
No counseling (*n*)		43	341			
Counseling (*n*)	Middle/high educational status	130	173	5.25 (3.60–7.65)	<0.001	>95.0%
No counseling (*n*)		50	351			

Counseling (*n*)	Age ≤ 30 yr	40	58	4.66 (2.71–8.01)	<0.001	>95.0%
No counseling (*n*)		30	190			
Counseling (*n*)	Age ≥ 31 yr	108	168	5.64 (3.90–8.14)	<0.001	>95.0%
No counseling (*n*)		56	509			

Counseling (*n*)	Parity = 1	67	100	4.46 (2.86–6.94)	<0.001	>95.0%
No counseling (*n*)		45	301			
Counseling (*n*)	Parity ≥ 2	87	120	5.91 (3.93–8.89)	<0.001	>95.0%
No counseling (*n*)		48	391			

ORs (95% CI) were estimated using logistic regression models adjusted for prepregnancy BMI, familial history of type 2 DM, previous GDM, parity. Post-hoc statistical power calculations were performed using G∗Power software 3.1, entering *R*-squared multiple correlation coefficient obtained with regression for each trait.

**Table:** (a)

	Followup (*n* = 374)	No followup (*n* = 785)	OR 1 (95% CI)	*P* value 1	Statistical power	OR 2 (95% CI)	*P* value 2
Age (≥31 yr versus ≤30 yr)	69 versus 305	174 versus 611	1.18 (0.89–1.58)	0.242	22.1%	1.27 (0.88–1.82)	0.198
Familiarity for type 2 DM (%)	270 (72.2)	535 (68.2)	1.21 (0.92–1.58)	0.178	31.6%	0.94 (0.68–1.31)	0.097
Prepregnancy BMI (kg/m^2^)							
<25 versus ≥25	174 versus 200	426 versus 359	1.41 (1.09–1.81)	0.008	81.5%	1.74 (1.27–2.37)	0.001
Previous GDM (%)	72 (19.3)	45 (5.7)	4.82 (3.17–7.33)	<0.001	>95.0%	5.30 (3.26–8.61)	<0.001
Parity (1 versus ≥2)	167 versus 207	346 versus 439	1.03 (0.80–1.32)	0.800	5.7%	1.11 (0.82–1.51)	0.500
Educational status							
Middle/high versus low	303 versus 71	401 versus 384	4.06 (3.02–5.45)	<0.001	>95.0%	3.54 (2.48–5.06)	<0.001
Insulin treatment (%)	147 (39.3)	253 (32.2)	2.32 (1.76–3.05)	<0.001	>95.0%	2.63 (1.90–3.66)	<0.001
PCOS (%)	72 (19.3)	37 (4.7)	5.27 (3.36–8.27)	<0.001	>95.0%	5.36 (3.24–8.85)	<0.001

OR 1: odd ratio after adjustment for any possible confounder; OR 2: As OR 1, but after stratification for absence of counseling; CI: confidence interval; Post-hoc statistical power calculations were performed using G∗Power software 3.1, entering *R*-squared multiple correlation coefficient obtained with regression for each trait. Low educational status: primary school; middle educational status: secondary school; high educational status: university degree; PCOS: polycystic ovary syndrome.

**Table:** (b)

	Followup (*n* = 374)	No followup (*n* = 785)	*P* value	Statistical power
Age (yr), mean ± SD	34.5 ± 5.2	33.1 ± 4.8	<0.001	>95.0%
Prepregnancy BMI (kg/m^2^), mean ± SD	25.9 ± 4.5	25.7 ± 4.7	0.265	67.4%
FPG (mg/dL), mean ± SD	92.8 ± 9.0	91.2 ± 10.7	0.027	>95.0%
Week at diagnosis (*n*), mean ± SD	25.3 ± 4.9	27.2 ± 3.4	<0.001	>95.0%

Continuous variables were compared using linear regression models adjusted for familial history of type 2 DM, parity, and prepregnancy BMI (when appropriate). Post-hoc statistical power calculations were performed using G∗Power software 3.1, entering partial *R*-squared obtained with regression for each trait. FPG: fasting plasma glucose; PCOS: polycystic ovary syndrome. All variables have been log-transformed to better approximate a normal distribution.
